# Optimized workup of lymph nodes in regard to UICC classification of colorectal carcinoma

**DOI:** 10.1111/his.70070

**Published:** 2026-01-07

**Authors:** Moritz Rust, Nabih Farkouh, Piet Beusker, Ali Eissing‐Al‐Mukahal, Clara Böker, Julian Mall, Ludwig Wilkens

**Affiliations:** ^1^ Institute of Pathology, Clinics of the Region Hannover Hanover Germany; ^2^ Clinic for Neurosurgery Justus‐Liebig University Gießen Germany; ^3^ Clinics of Visceral and Minimal‐Invasive Surgery Clinics of the Region Hannover Hanover Germany; ^4^ Institute of Human Genetics, Medical School Hannover Hanover Germany

## Abstract

**Aims:**

Treatment and outcomes in colorectal carcinoma (CRC) depend on the UICC classification, depth of invasion, and distant metastases. However, it is not clear whether an extensive workup of lymph nodes (LNs) is important for the adequate determination of metastases. Therefore, we compared the number of LNs and metastases obtained by a standard protocol (SP) and an extended protocol (EP) in two series with a total of 105 CRC cases and 2417 LNs.

**Methods and results:**

In the first series, the EP included complete stepwise sectioning of all LN‐bearing paraffin blocks in each case, increasing the total number of LNs from 1247 in the SP to 1333 in the EP and the number of metastases from 69 to 80. One pTNM pN1a case became pN1b, and two pN1b cases became pN2a. The staging, and thus the therapy, changed in none of them. Furthermore, no relevant effect of embedding one versus both halves of large LNs >5 mm was evident. In the second series, the EP included immunohistochemical staining for pan‐cytokeratin (monoclonal antibodies AE1/AE3) of all LN‐bearing paraffin blocks in a given case. The number of detected metastases rose from 82 to 89, with a constant total 1084 LNs. In two cases, the pTNM classification changed from pN2a to pN2b. Staging and therapy changed in none.

**Conclusion:**

An extensive workup of LNs is not mandatory in patients with CRC. A straightforward protocol is sufficient to guide clinicians to the appropriate therapy.

## Graphical abstract

Treatment and outcome of colon cancer depend significantly on the histopathological examination of lymph nodes. Methodologically, stepwise sectioning and immunohistochemistry are the primary methods used. The extent of this examination is subject to debate but should be maintained at an adequate level in routine laboratory work.

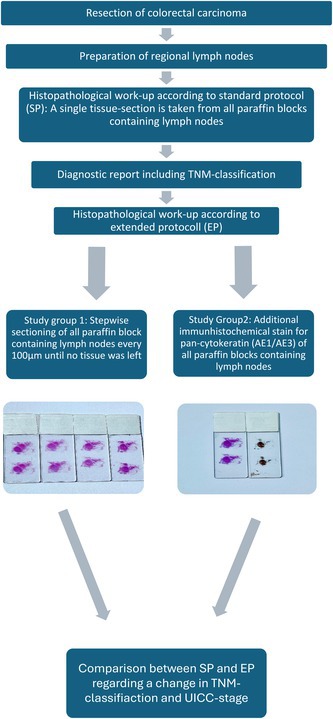

**Abbreviations**
CRC: colorectal carcinoma
EP: extended protocol
ITC: isolated tumour cells
LNs: lymph nodes
SD: standard deviation
SP: standard protocol

## Introduction

The incidence of colorectal carcinoma (CRC) has increased in recent years.^1^ Approximately, 10% of all cancer deaths are currently caused by CRC. With increasing tumour size, the risk of metastases in lymph nodes (LNs) also increases.^2^, ^3^ The number of LN metastases in CRC is a major factor in treating patients^4^, ^5^, ^6^, ^7^, ^8^, ^9^, ^10^, ^11^, ^12^, ^13^, ^14^, ^15^, ^16^, ^17^, ^18^ and one of the three main factors considered in the UICC classification (8th edition). Having fewer than 12 LNs examined is reported to be a risk factor regardless of the presence of metastases.^19^, ^20^ Accordingly, the American Joint Committee on Cancer has set a minimum of 12 LNs as adequate for UICC classification.^21^ Therefore, it is essential to carefully search for metastases in the gross and histological examinations.

Gross pathology techniques such as acetone clearing^22^, ^23^ or pretreatment with sodium hypochlorite^24^ have been demonstrated to be helpful in increasing the number of LNs harvested in CRC specimens, so that the number of LNs (12) necessary for adequate staging^25^ is reached more often. The probability of detecting metastasis in a given LN depends on the histological technique and effort spent.^8^ This has been shown in LNs collected from patients with malignant melanoma.^26^ However, an exhaustive workup of LNs in melanoma is not necessary to achieve adequate results.^27^

To the best of our knowledge, no studies have found an optimal balance between identifying metastases and the effort necessary to achieve this. This is surprising because histological examination of LNs for metastases is a significant part of the daily work of a pathologist. Furthermore, these examinations are not standardized.^28^, ^29^, ^30^ The aim of our study was to investigate which histological protocol is appropriate for defining the number of LN metastases in a CRC sample. For this purpose, we analysed 1333 LNs by complete serial sectioning and compared the results with the findings from a standard protocol. We used a similar approach to investigate the utility of immunohistochemistry in searching for metastases in a different group of 1084 LNs.

## Material and Methods

Surgical specimens from 105 patients with CRC were included in this prospective study and divided into two groups (Table 1). Mesenteric and/or pararectal fat was taken from all specimens and prepared by slicing the tissue into 3‐mm sections, taking out visible LNs, followed by paraffin embedding. Detectable LNs were treated in two different ways in each group, named the standard protocol (SP) and the extended protocol (EP).

**Table 1:** Clinicopathological data

Case	Age	Sex	Localization (ICD‐O‐3)	Tumour diameter	Histological classification (ICD‐O‐3)	Histological grade	pTNM/cTNM	R‐status
*Group 1*								
1	98	F	C18.0	48	M8140/3	3	pT4b pN0 cM0 L0 V0 R0	R0
2	72	F	C18.6	21	M8480/3	2	pT3 pN1b cM0 L1 V1 R0	R0
3	83	F	C18.2	22	M8140/3	3	pT2 pN0 cM0 L0 V0 R0	R0
4	60	M	C18.2	32	M8140/3	2	pT3 pN2b cM0 L1 V1 R0	R0
5	84	F	C19	45	M8140/3	2	pT3 pN1a cM0 L0 V0 R0	R0
6	78	M	C20	45	M8140/3	2	pT3 pN1b cM0 L0 V0 R0	R0
7	79	F	C19	37	M8140/3	2	pT2 pN1a cM0 L0 V0 R0	R0
8	87	F	C18.7	32	M8140/3	2	pT3 pN0 cM0 L0 V0 R0	R0
9	83	M	C18.2	30	M8140/3	3	pT3 pN0 cM0 L1 V0 R0	R0
10	70	M	C18.2	50	M8140/3	2	pT3 pN0 cM0 L0 V0 R0	R0
11	68	M	C18.0	55	M8140/3	2	pT3 pN0 cM0 L0 V0 R0	R0
12	36	F	C18.7	45	M8140/3	2	pT3 pN1b cM0 L1 V0 R0	R0
13	83	M	C18.0	48	M8140/3	2	pT3 pN0 cM0 L0 V0 R0	R0
14	96	F	C18.0	72	M8020/3	3	pT4b pN2b cM0 L0 V0 R0	R0
15	82	F	C18.2	40	M8140/3	3	pT3 pN0 cM0 L1 V0 R0	R0
16	76	M	C18.2	35	M8140/3	2	pT3 pN0 cM0 L0 V0 R0	R0
17	72	F	C18.6	45	M8140/3	2	pT3 pN0 cM0 L0 V0 R0	R0
18	69	F	C18.4	45	M8140/3	2	pT3 pN0 cM0 L0 V0 R0	R0
19	65	M	C20	48	M8140/3	3	pT3 pN1b cM0 L0 V0 R0	R0
20	62	M	C19	20	M8140/3	3	pT2 pN0 cM0 L0 V0 R0	R0
21	62	F	C18.4	44	M8246/3	3	pT4a pN2a pM1 L1 V1 R0	R0
22	71	M	C18.2	37	M8140/3	2	pT1 pN0 cM0 L0 V0 R0	R0
23	71	M	C18.3	45	M8140/3	2	pT2 pN0 cM0 L0 V0 R0	R0
24	65	M	C18.3	65	M8140/3	2	pT3 pN0 cM0 L0 V0 R0	R0
25	55	M	C18.7	25	M8140/3	2	pT2 pN0 cM0 L0 V0 R0	R0
26	85	F	C18.2	22	M8140/3	3	pT4a pN1c cM0 L1 V0 R0	R0
27	82	F	C18.4	63	M8140/3	3	pT4b pN1b cM0 L0 V0 R0	R0
28	84	M	C19	45	M8140/3	3	pT3 pN2b cM0 L1 V0 R0	R0
29	79	F	C18.7	45	M8140/3	2	pT3 pN0 cM0 L1 V0 R0	R0
30	82	M	C18.0	28	M8140/3	2	pT2 pN0 cM0 L0 V0 R0	R0
31	85	F	C18.0	52	M8140/3	3	pT3 pN1b cM0 L0 V0 R0	R0
32	53	M	C18.0	17	M8140/3	3	pT3 pN2a cM0 L1 V0 R0	R0
33	58	M	C18.0	37	M8140/3	2	pT3 pN0 cM0 L0 V0 R0	R0
34	51	F	C18.2	50	M8140/3	3	pT3 pN1b cM1 L1 V1 R0	R0
35	71	M	C18.6	95	M8140/3	3	pT3 pN1b cM0 L0 V0 R0	R0
36	80	M	C18.0	27	M8140/3	3	pT3 pN1b cM0 L1 V1 R0	R0
37	61	M	C19	45	M8140/3	2	pT4a pN1b cM0 L1 V0 R0	R0
38	78	F	C18.3	17	M8140/3	2	pT2 pN0 cM0 L0 V0 R0	R0
39	81	M	C18.2	48	M8140/3	2	pT3 pN0 cM0 L0 V0 R0	R0
40	84	M	C18.0	32	M8140/3	2	pT3 pN0 cM0 L1 V0 R0	R0
41	75	M	C18.4	52	M8140/3	2	pT3 pN1a cM0 L1 V0 R0	R0
42	79	F	C18.2	22	M8480/3	2	pT1 pN0 cM0 L0 V0 R0	R0
43	75	M	C18.4	48	M8140/3	2	pT3 pN1b cM1a L0 V1 R0	R0
44	74	F	C18.2	42	M8140/3	2	pT3 pN0 cM0 L0 V0 R0	R0
45	57	F	C18.3	28	M8140/3	2	pT3 pN0 pM1a L1 V0 R0	R0
46	79	M	C18.4	44	M8140/3	2	pT2 pN0 cM0 L0 V0 R0	R0
47	61	M	C18.7	62	M8140/3	2	pT4b pN1a pM1a L1 V0 R0	R0
48	62	M	C20	26	M8140/3	2	pT2 pN0 cM0 L0 V0 R0	R0
49	77	M	C18.0	55	M8140/3	2	pT3 pN0 cM0 L0 V0 R0	R0
50	43	M	C18.2	52	M8140/3	2	pT3 pN0 cM0 L0 V0 R0	R0
*Group 2*								
1	69	M	C18.8	12	M8140/4	3	pT1 pN0 cM0 L0V1	R1
2	72	F	C20	50	M8140/3	2	pT3 pN0 cM0 L0V0	R0
3	45	M	C18.6	70	M8140/3	2	pT1 pN0 cM0 L0V0	R0
4	71	M	C18.7	65	M8140/3	2	pT4a pN0 cM0 L0V0	R0
5	76	M	C20	50	M8140/3	2	pT3 pN0 cM0 L0V0	R0
6	61	M	C18.0	40	M8140/3	2	pT2 pN0 cM0 L0V0	R0
7	74	F	C18.2	90	M8140/3	2	pT3 pN0 cM0 L0V0	R0
8	83	F	C20	35	M8140/3	2	pT3 pN0 cM0 L0V0	R0
9	73	F	C18.7	53	M8140/3	2	pT3 pN0 cM0 L0V0	R0
10	55	M	C18.7	19	M8140/3	2	pT2 pN0 cM0 L0V0	R0
11	85	F	C18.4	33	M8140/3	2	pT3 pN0 cM0 L0V0	R0
12	83	M	C18.5	60	M8140/3	2	pT3 pN0 cM0 L1V0	R0
13	67	M	C18.7	59	M8140/3	2	pT3 pN0 cM0 L0V0	R0
14	83	M	C18.7	25	M8140/3	2	pT1 pN0 cM0 L0V0	R0
15	82	F	C18.0	70	M8140/3	2	pT3 pN0 cM0 L0V0	R0
16	83	M	C18.0	48	M8140/3	2	pT3 pN0 cM0 L0V0	R0
17	85	F	C18.0	75	M8140/3	2	pT3 pN0 cM0 L0V0	R0
18	72	M	C18.0	110	M8140/3	3	pT4b pN0 cM0 L0V0	R0
19	69	M	C18.2	6	M8140/3	2	pT1 pN0 cM0 L0V0	R0
20	91	M	C18.2	64	M8140/3	2	pT4a pN0 cM0 L0V0	R0
21	81	F	C18.2	54	M8140/3	2	pT3 pN0 cM0 L0V0	R0
22	73	M	C18.3	93	M8140/3	2	pT3 pN0 cM0 L0V0	R0
23	69	F	C18.2	80	M8140/3	2	pT3 pN0 cM0 L0V0	R0
24	74	M	C18.2	90	M8140/3	3	pT3 pN0 cM0 L0V0	R0
25	82	M	C18.2	60	M8140/3	2	pT2 pN0 cM0 L0V0	R0
26	50	M	C19	6	M8140/3	2	pT1 pN0 cM0 L0V0	R0
27	86	M	C18.2	33	M8140/3	2	pT2 pN0 cM0 L0V1	R0
28	74	F	C19	65	M8140/3	2	pT4a pN0 cM0 L0V0	R0
29	67	M	C18.6	28	M8140/3	2	pT3 pN0 cM0 L0V0	R0
30	76	F	C18.6	75	M8140/3	2	pT3 pN0 cM0 L0V0	R0
31	67	M	C18.7	25	M8140/3	2	pT2 pN1a cM0 L1V0	R0
32	80	F	C18.4	98	M8140/3	3	pT3 pN1a cM0 L0V0	R0
33	90	F	C19	29	M8140/3	2	pT3 pN1a cM0 L1V0	R0
34	86	F	C18.2	58	M8140/3	3	pT3 pN1a cM0 L1V0	R0
35	60	M	C19	26	M8140/3	2	pT3 pN1b cM0 L1V1	R0
36	75	F	C18.3	100	M8140/3	2	pT4b pN1b cM0 L0V0	R0
37	81	M	C18.0	68	M8140/3	2	pT3 pN1b cM0 L1V0	R0
38	76	M	C18.7	35	M8140/3	2	pT3 pN1b cM0 L1V1	R0
39	74	M	C20	33	M8140/3	2	pT3 pN1b cM0 L0V1	R0
40	73	F	C18.2	58	M8140/3	2	pT3 pN1b cM0 L0V0	R0
41	55	M	C20	55	M8480/3	2	pT4a pN1b cM0 L1V0	R0
42	68	M	C18.0	65	M8140/3	2	pT3 pN1b pM1a L1V0	R0
43	69	F	C18.7	42	M8140/3	2	pT3 pN1b cM1a L1V0	R0
44	69	F	C18.0	56	M8140/3	2	pT3 pN1b cM0 L0V0	R0
45	81	M	C18.0	125	M8480/3	2	pT4b pN1b cM0 L1V0	R0
46	77	M	C18.2	48	M8140/3	2	pT3 pN2a cM0 L1V1	R0
47	62	F	C18.0	40	M8140/3	2	pT4b pN2a pM1c L1V0	R2
48	79	F	C18.7	75	M8140/3	2	pT2 pN2a pM1a L1V0	R2
49	76	F	C18.3	31	M8140/3	2	pT3 pN2a cM0 L0V0	R0
50	73	M	C18.7	42	M8140/3	2	pT3 pN2a cM0 L0V0	R0
51	80	M	C18.4	60	M8154/3	3	pT3 pN2a cM0 L1V0	R0
52	80	M	C18.0	41	M8140/3	2	pT2 pN2a pM1a L1V0	R0
53	69	M	C18.7	30	M8140/3	2	pT3 pN2a cM0 L0V0	R0
54	90	M	C18.2	75	M8140/3	2	pT3 pN2b cM0 L1V0	R0
55	61	M	C20	45	M8140/3	2	pT3 pN2b cM0 L0V0	R0

This study was registered by the Ethic committee of the Medical school Hanover under number 11117BO‐K‐2023.

### Group 1—Standard Protocol

Fifty cases of CRC were included. For the SP examination, LNs with a diameter ≤5 mm were placed whole in cassettes and paraffin embedded using standard techniques. LNs >5 mm were cut into equal halves and embedded in different cassettes (A and B). The A cassettes were used for the SP (and after this also worked up by using EP) and the B cassettes for the EP as described below. Each cassette had no more than three LNs, regardless of size. In addition, all examined LNs were located in the D1 compartment and grouped on the basis of the distance between the LN and the carcinoma. LNs within 5 cm of the tumour were considered ‘near the tumor’, whereas LNs >5 cm from the tumour were ‘distant from the tumor’. From among all the paraffin blocks, one 3‐μm tissue section was taken and stained with H&E. Histological examination was carried out by the pathologists responsible for these particular cases in daily routine work.

### Group 1—Extended Protocol

After the SP was finished and a report signed out, the EP was carried out for all paraffin‐embedded LNs, including those in the B cassettes. From all paraffin blocks, stepped sections were made every 100 μm until no tissue was left. Histological examination was carried out by three experienced pathologists (M.R., N.F., and L.W.).

### Group 2—Standard Protocol

Fifty‐five cases of CRC were included. All detected LNs were placed intact in cassettes and paraffin embedded using standard techniques, regardless of size or location relative to the tumour. Afterwards, the routine procedure was done as described above (Group 1‐SP).

### Group 2—Immuno Protocol

After the SP was finished and a report signed out, all paraffin blocks containing LNs received an immunohistochemical stain for pan‐cytokeratin (AE1/AE3).^31^, ^32^, ^33^ Histological examination was carried out by three experienced pathologists (M.R., N.F., and L.W.).

On the basis of the number of LNs and metastases detected by the SP and EP, statistical analysis was carried out in regard to particular aspects of the results, which are reported in more detail below. These results were compared with the pTNM classification and staging given in the 9th edition of the UICC classification.

To begin the analysis of the mean numbers of LNs and metastases, we performed a Kolmogorov–Smirnoff test with a Lilliefors correction of the level of significance. This allowed us to determine whether both data sets are normally distributed. For both variables (i.e., LNs/metastases in SP and LNs/metastases in EP), there was a probability error with *P* < 0.001. Therefore, the hypothesis that both data sets are normally distributed has to be rejected. For the comparison of the mean numbers of metastases, we performed the Wilcoxon rank sum test as a non‐parametric test of connected samples.

The level of significance for one‐sided testing of the hypothesis was set at *ɑ* = 0.025.

It should be noted that the count of metastases was determined strictly by the rules of TNM classification, which mandate the inclusion of all intranodal tumour deposits of size ≥0.2 mm. However, intranodal tumour deposits <0.2 mm in size and subserosal tumour deposits without evidence of residual lymph node structure were excluded.

## Results

To provide a better overview, the results are given in four parts:

In part I, the absolute numbers of LNs and metastases in study Group 1 determined by the SP and EP were analysed with regard to five aspects. In part II, we focused on the local distribution of the LNs in the surgical specimens of study Group 1.c In part III, we evaluated the effect of immunohistochemistry on searching for metastases in study Group 2. In part IV, we looked at the material and time spent for the approach used in study Group 1.

### Part I: Effect of Stepwise Sectioning

The number of LNs changed significantly when the EP was used. Using the SP, the total number of LNs was 1247. The mean number of LNs per patient was 24.94 with a standard deviation (SD) of 9.85. Using the EP, a higher number of LNs was counted: 1.333 (6.9% more than the SP‐derived number). The mean number of LNs per patient was 26.66 (SD 10.89). These additional LNs were not macroscopically visible at the time of embedding, but were discovered microscopically at deeper cutting levels. The difference in the mean number of LNs was 1.72 (*P* < 0.001).

The number of detected metastases changed significantly when the EP was used. In the SP, 21 patients had 69 metastases. The mean number of metastases per patient (*n* = 50) was 1.38 (range 1–11; SD 2.26). In the EP, 80 metastases were detected in 21 patients. The mean number of metastases per patient was 1.60 (range 1–11; SD 2.66). The difference in the number of metastases was 0.22 (*P* < 0.001).

The significant increase in the number of metastases did not depend exclusively on the complete sectioning of the embedded LNs but also on increasing the number of LNs. Next, we looked for the reason why more metastases were detected by the EP. Separating the macroscopically harvested and histologically presented LNs is important. Regarding the same 1247 LNs, the mean number of detected metastases was 1.38 by the SP and 1.54 by the EP. This difference of 0.16 was not significant (*P* = 0.0938). The metastases in the additionally detected LNs account for the difference.

A subgroup analysis supports this evidence: in cases with a constant number of LNs, the mean number of metastases detected by the SP was 1.05, and by the EP, 1.14. The difference was 0.09 (*P* = 0.157). In cases with a variable number of LNs, the mean number of metastases detected by the SP was 1.62, and by the EP, 1.93. The difference was 0.31 (*P* = 0.014).

Embedding both halves of large LNs did not change the staging. As noted above, there were 11 additional metastases found after using the EP. Three of these metastases occurred in LNs with a diameter >5 mm. In one of these LNs, a metastasis was found when stepwise sectioning was performed for the half embedded in the A cassette without finding a metastasis in the B cassette. Two metastases were seen when stepwise sectioning the B cassettes but were not the A cassettes. Thus, all of these three metastases were seen in only one half of the LN. These additional metastases did not change the UICC stage and, therefore, did not change the patients' treatment.

The increased number of metastases led to changes in the pTNM classification but did not influence disease stage or treatment. In nine cases, the EP led to an increase in metastases (Tables 2 and 3). In six of these cases, no changes were seen in the pTNM classification. In three of these cases, a change was seen: in two cases, the pN category switched from pN1b to pN2a, and in one case, the pN category switched from pN1a to pN1b. None of these cases had a change in the stage. Accordingly, there was also no change in the treatment.

**Table 2:** Raw data for all cases examined

Group 1: SP versus EP			Group 2: SP versus EP	
Case number	Number of LNs SP/EP/difference	Number of metastases SP/EP/difference	Number of LNs. SP	Number of metastases SP/EP/difference
1	22/22/0	0/0/0	14	0/0/0
2	29/33/4	3/5/2	13	0/0/0
3	25/25/0	0/0/0	23	0/0/0
4	24/30/6	11/11/0	15	0/0/0
5	25/27/2	1/1/0	19	0/0/0
6	23/23/0	3/3/0	14	0/0/0
7	17/20/3	1/1/0	23	0/0/0
8	18/20/2	0/0/0	18	0/0/0
9	16/18/2	0/0/0	14	0/0/0
10	26/31/5	0/0/0	16	0/0/0
11	19/19/0	0/0/0	27	0/0/0
12	39/54/15	3/3/0	16	0/0/0
13	21/21/0	0/0/0	21	0/0/0
14	23/24/1	8/9/1	14	0/0/0
15	38/38/0	0/0/0	17	0/0/0
16	31/32/1	0/0/0	20	0/0/0
17	31/33/2	0/0/0	32	0/0/0
18	39/41/2	0/0/0	26	0/0/0
19	23/23/0	2/2/0	23	0/0/0
20	16/16/0	0/0/0	20	0/0/0
21	60/60/0	3/3/0	18	0/0/0
22	22/22/0	0/0/0	16	0/0/0
23	19/20/1	0/0/0	18	0/0/0
24	18/18/0	0/0/0	20	0/0/0
25	15/15/0	0/0/0	20	0/0/0
26	15/15/0	1/1/0	17	0/0/0
27	20/21/1	2/3/1	20	0/0/0
28	38/43/5	8/10/2	22	0/0/0
29	17/18/1	0/0/0	17	0/0/0
30	20/20/0	0/0/0	18	0/0/0
31	18/18/0	0/2/0	30	1/1/0
32	18/18/0	5/6/1	29	1/1/0
33	26/31/5	0/0/0	23	1/1/0
34	21/21/0	2/3/1	23	1/1/0
35	24/24/0	3/3/0	13	3/3/0
36	36/44/8	3/4/1	19	2/2/0
37	23/24/1	3/3/0	28	3/3/0
38	19/23/4	0/0/0	18	2/2/0
39	25/27/2	0/0/0	13	2/2/0
40	21/22/1	0/0/0	17	2/3/1
41	16/17/1	1/1/0	17	3/3/0
42	19/19/0	0/0/0	31	2/2/0
43	51/53/2	2/3/1	17	2/2/0
44	18/19/1	0/0/0	18	2/2/0
45	48/48/0	0/0/0	22	2/2/0
46	18/19/1	0/0/0	14	4/5/1
47	29/33/4	1/2/1	13	4/5/1
48	14/15/1	0/0/0	35	4/4/0
49	20/22/2	0/0/0	18	5/5/0
50	34/34/0	0/0/0	16	4/6/2
51	xxx	xxx	17	4/4/0
52	xxx	xxx	21	6/7/1
53	xxx	xxx	15	6/6/0
54	xxx	xxx	20	9/9/0
55	xxx	xxx	26	7/7/0
	1247/1333	69/80/11	1084	82/88/6
Mean SP/EP/difference	24.94/26.66	1.38/1.6	19.71	1.49/1.62
Standard dev. SP/EP	9.85/10.89	2.36/2.66	5.25	2.12/2.32

**Table 3:** Summary of all cases with an increased number of metastases using SP/EP. Overall, EP did not change the staging in any case

Case	SP			EP			SP versus EP	
	LN status	pTNM/cTNM	Staging	LN status	pTNM/cTNM	Staging	Change in pTNM	Change in staging
2	3/29	T3/N1b/M0	III B	5/33	T3/N2a/M0	III B	Yes	No
14	8/23	T4b/N2b/M0	III C	9/24	T4b/N2b/M0	III C	No	No
27	2/20	T4b/N1b/M0	III C	3/21	T4b/N1b/M0	III C	No	No
28	8/38	T3/N2b/M0	III C	10/43	T3/N2b/M0	III C	No	No
32	5/18	T3/N2a/M0	III B	6/18	T3/N2a/M0	III B	No	No
34	2/21	T3/N1b/M1c	IV C	3/21	T3/N1b/M1c	IV C	No	No
36	3/36	T3/N1b/M0	III B	4/44	T3/N2a/M0	III B	Yes	No
43	2/51	T3/N1b/M1a	IV A	3/53	T3/N1b/M1a	IV A	No	No
47	1/19	T4b/N1a/M1a	IV A	2/33	T4b/N1b/M1a	IV A	Yes	No

Case	SP			IP			SP versus IP	
	LN status	pTNM/cTNM	Staging	LN status	pTNM/cTNM	Staging	Change in pTNM	Change in staging
40	2/17	T3/N1b/M0	III B	3/17	T3/N1b/M0	III B	No	No
46	4/14	T3/N2a/M0	III B	5/14	T3/N2a/M0	III B	No	No
47	4/13	T4b/N2a/M1c	IV C	5/13	T4b/N2a/M1c	IV C	No	No
50	4/16	T3/N2a/M0	III B	6/16	T3/N2a/M0	III B	Yes	No
52	6/21	T2/N2a/M1a	IV A	7/21	T2/N2b/M1a	IV A	Yes	No

To complete our analysis of group 1, we also looked for differences in regard to the total number of lymphnodes analysed. This was done to exclude an effect on the results due to a higher number of lymphnodes, caused probably by a more intense search for lymphnodes in gross examination. For this analysis, we parted group 1 in two subgroups with less than 20 or more than 19 lymphnodes. By this we saw <20 lymphnodes in 22 cases of group 1. In these 22 cases, there were 366 lymphnodes (mean of 16.6/case) and 21 metastases (mean of 0.95 metastases/case). In one case (patient 32), there was a single additional metastasis in EP. The lymphnode bearing this metastasis was seen in gross examination and SP examination, respectively. Beside this metastasis four other metastases were seen in lymphnodes already found in gross examination and SP. This did not change pTNM classification or staging, respectively. Therefore the absolute number of lymphnodes examined had no impact on the overall results.

### Part II: Local Distribution of Metastases

**Figure 1:**
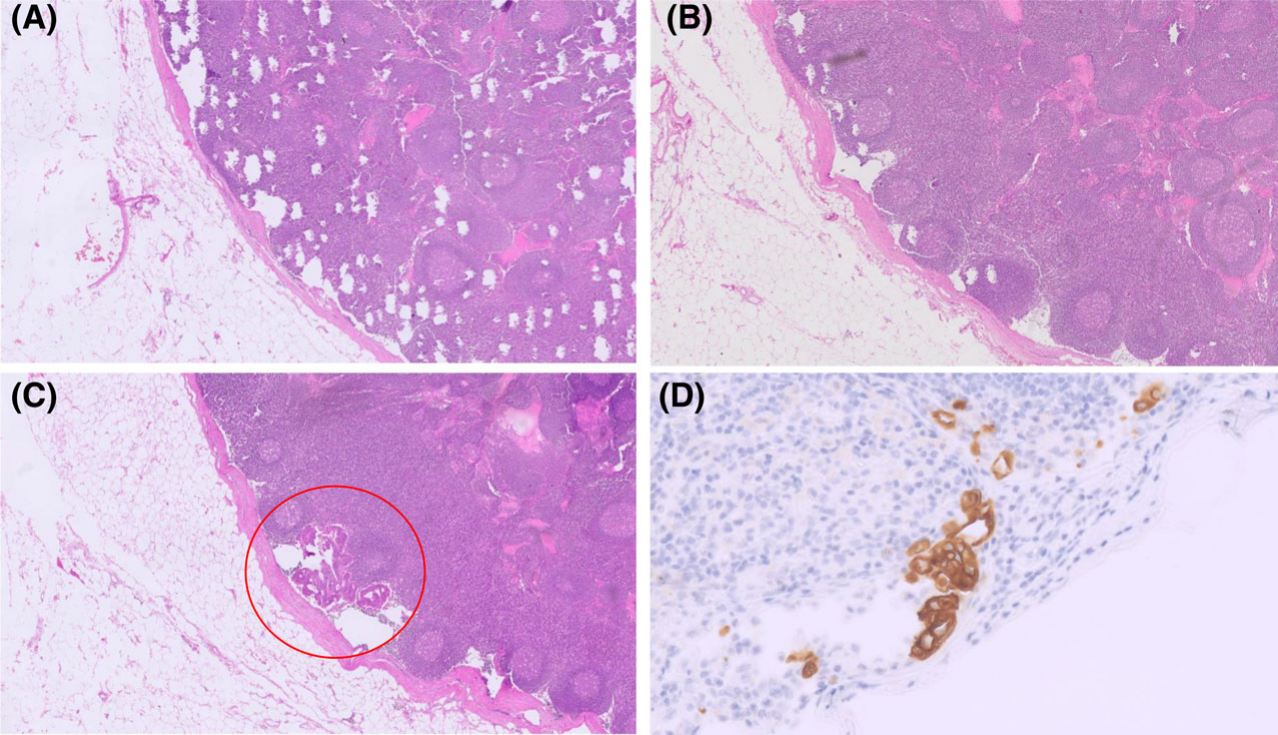
(**A–C**) Metastasis in a lymphnode found by EP only. Whereas tissue sections 1–12 (**A**: section1, **B**: section 5, original magnification 10X5) were free of metastasis, in tissue sections 13 (**C**) and following a metastasis occured (red circle). (**D**) isolated tumour cells (ITC) in a lymphnode, only detected by immunhistological staining with antibody panCK (original magnification: 10 × 40).

The distance of LN metastases from the tumour does not play a special role in the UICC classification. Nevertheless, we wanted to know where metastases were most commonly located and where the additional metastases found by the EP occurred. Therefore, we defined LNs and metastases as near the tumour (≤50 mm) and distant from the tumour (>50 mm), as shown in Figure 1.

The SP revealed 69 metastases, including 66 near the tumour and three distant from the tumour. The EP revealed 11 additional LNs, 8 of them near the tumour and three distant from the tumour (Table 4). The differences between the LN–tumour distances in the SP and EP were significant (*P* < 0.001, tested for symmetrical binomial distribution).

**Table 4:** Number of LNs and metastases with a distance ≤50 mm or >50 mm from the tumour (based on data of Group 1)

Case	LNs			Metastases		
	Number of LNs	≤50 mm/>50 mm	% of near LNs	Number of metastases	≤50 mm/>50 mm	% of near metastases
1	22	6/16	27	0	0/0	
2	33	13/20	39	5	4/1	80
3	25	11/14	44	0	0/0	
4	30	17/13	57	11	11/0	100
5	27	6/21	22	1	1/0	100
6	23	19/4	83	3	3/0	100
7	20	17/3	85	1	1/0	100
8	20	14/6	70	0	0/0	
9	18	11/7	61	0	0/0	
10	31	21/10	68	0	0/0	
11	19	14/5	74	0	0/0	
12	54	28/26	52	3	3/0	100
13	21	17/4	81	0	0/0	
14	24	19/5	79	9	9/0	100
15	38	20/18	53	0	0/0	
16	32	11/21	34	0	0/0	
17	33	10/23	30	0	0/0	
18	41	15/26	37	0	0/0	
19	23	15/8	65	2	2/0	100
20	16	6/10	38	0	0/0	
21	60	20/40	33	4	3/1	75
22	22	11/11	50	0	0/0	
23	20	9/11	45	0	0/0	
24	18	11/7	61	0	0/0	
25	15	11/4	73	0	0/0	
26	15	9/6	60	1	1/0	100
27	21	11/10	52	3	1/2	33
28	43	32/11	74	10	10	100
29	18	15/3	83	0	0/0	
30	20	16/4	80	0	0/0	
31	18	5/13	28	2	2/0	100
32	18	10/8	56	6	6/0	100
33	31	29/2	94	0	0/0	
34	21	18/3	86	3	3/0	100
35	24	18/6	75	3	3/0	100
36	44	25/19	57	4	4/0	100
37	24	22/2	92	3	3/0	100
38	23	6/17	26	0	0/0	
39	27	14/13	52	0	0/0	
40	22	18/4	82	0	0/0	
41	17	6/11	35	1	1/0	100
42	19	4/15	21	0	0/0	
43	53	32/21	60	3	3/0	100
44	19	7/12	37	0	0/0	
45	48	20/28	42	0	0/0	
46	19	8/11	42	0	0/0	
47	33	14/19	42	2	1/1	50
48	15	8/7	53	0	0/0	
49	22	8/14	36	0	0/0	
50	34	14/20	41	0	0/0	
	Total	≤50 mm	% of near LNs	Total	≤50 mm	% of near metastases
	1333	721	54.1^*^	80	75	93.8^*^

* In regard to the distance no significant difference was found in the number of LNs whereas the number of metastases, respectively, was highly significant (*P* < 0.00015).

Overall, we found 80 metastases by using the EP: 75 near the tumour (94%) and five distant from the tumour (6%). These distant metastases were seen in patients 2, 21, 27, and 47. The pTNM classification was upgraded due to additional distant metastases, with a shift from pN1b to pN2a in Patient 2. The classification was shifted from pN1a to pN1b in patient 47 due to one additional distant and one additional near metastasis. In all of these patients, there were also metastases near the tumour. In no patient did the staging or clinical treatment change.

### Part III: Effect of Immunohistochemistry

The number of detected metastases changed significantly when the EP was used. In 25 patients, the SP detected 82 metastases. The mean number of metastases per patient (*n* = 55) was 1.49 (range 1–9; SD 2.12). In 25 patients, the IP detected 88 metastases. The mean number of metastases per patient was 1.62 (range 1–9; SD 2.32). The difference in the mean number of metastases was 0.13 (*P* = 0.034).

In five cases, the EP detected more metastases. One of these cases shifted from pN2a to pN2b without change in staging. However, it has to be mentioned, that in a further lymph node ITCs were seen, not regarded as a metastasis in accordance to UICC classification. In the other four cases, the pTNM classification did not change.

### Part IV: Time of Extensive Workup

The time needed to perform a histopathological workup may vary from laboratory to laboratory. To avoid overcomplicating this analysis, we assumed the following required times based on our local conditions:

- About 60 s for a tissue section to be processed manually by a medical‐technical assistant.
- About 30 s for a tissue section to be examined microscopically by a pathologist.

After performing SP and EP on study Group 1, we were able to draw a comparison (Table 5).

**Table 5:** Time needed for histological examination using EP versus SP (based on data of Group 1)

Case	SP			EP				SP versus EP
	Total number of slides	Slides used for LNs	Time needed, (min)^a^	Total number of slides	Slides used for LNs	Additional tissue sections	Time needed, (min)	Additional time needed (min)
1	74	34	37	247	207	575	325	288
2	19	10	10	73	64	130	75	65
3	33	26	17	123	116	261	147	131
4	33	25	17	210	202	419	226	210
5	42	30	21	257	245	518	280	259
6	20	14	10	94	88	183	102	92
7	21	15	11	128	122	226	124	113
8	35	18	18	117	100	259	147	130
9	19	13	10	115	109	228	124	114
10	32	23	16	208	199	408	220	204
11	22	14	11	142	134	275	149	138
12	37	28	19	243	234	486	262	243
13	44	31	22	209	196	410	227	205
14	34	17	17	161	144	312	173	156
15	37	29	19	208	200	399	218	200
16	36	24	18	183	171	335	186	168
17	29	21	15	142	134	296	163	148
18	43	31	22	347	335	668	356	334
19	28	21	14	148	141	289	159	145
20	23	13	12	98	88	113	68	57
21	38	23	19	131	116	249	144	125
22	27	19	14	141	133	263	145	132
23	26	19	13	144	137	273	150	137
24	28	18	14	132	122	247	138	124
25	20	12	10	90	82	174	97	87
26	24	13	12	80	69	157	91	79
27	28	14	14	125	111	243	136	122
28	42	31	21	276	265	550	296	275
29	19	12	10	78	71	149	84	75
30	19	10	10	75	66	140	80	70
31	25	15	13	153	143	302	164	151
32	25	16	13	154	145	284	155	142
33	38	24	19	339	325	664	351	332
34	24	16	12	103	95	195	110	98
35	30	19	15	129	118	243	137	122
36	41	28	21	299	286	576	309	288
37	32	19	16	186	173	367	200	184
38	20	11	10	82	73	157	89	79
39	33	21	17	153	141	287	160	144
40	35	18	18	141	124	259	147	130
41	31	14	16	116	99	210	121	105
42	39	16	20	109	86	206	123	103
43	31	19	16	155	143	313	172	157
44	28	15	14	123	110	234	131	117
45	23	12	12	73	62	140	82	70
46	29	16	15	118	105	228	129	114
47	36	22	18	192	178	353	195	177
48	22	10	11	80	68	151	87	76
49	25	15	13	129	119	241	133	121
50	46	32	23	222	208	426	236	213
Mean	31	19	15	156	144	301	166	151
SD	10	7	5	67	66	138	73	69
					Additional time needed per case (in h; mean/SD)			2:31/1:09

^a^ Calculation was based on a mean time of 30 s for histological examination of a tissue section.

The mean number of slides with LN tissue sections was 19 in the SP and 301 in the EP. The mean processing times for LN tissue sections were 0.32 h in the SP and 5.02 h in the EP. Examining LNs took a mean time of 0.16 h in the SP, but 2.51 h in the EP.

## Discussion

The UICC classification is a fundamental part of determining the treatment of patients with malignant neoplasms. The 8th edition (2016) is commonly accepted, with depth of invasion, number of LNs, and metastasis as the important factors. Therefore, an adequate number of LNs is crucial to determining a correct pTNM classification. Pathologists routinely find that identifying 12 LNs, the minimal number of LNs for classifying CRC,^21^ is time consuming and not achievable for all patients.^34^, ^35^, ^36^ In addition, focused, continuous attention is needed to do this work properly. Pretreatment of fat is helpful. Acetone treatment of the prepared fat has been shown to increase the number of detected LNs, making it possible to find 12 LNs in nearly all cases.^23^

Nevertheless, it seems very difficult to define ‘appropriate’ in regard to the histological examination.

The 8th edition does not mention how particular results used for the UICC classification were obtained. Therefore, some may argue that all LNs have to be completely worked up by sectioning to avoid missing metastases.^37^ This approach is reflected in many examinations from the last decades concerning micrometastases and/or isolated tumour cells in colorectal cancer.^38^, ^39^, ^40^, ^41^, ^42^, ^43^, ^44^, ^45^, ^46^, ^47^, ^48^, ^49^, ^50^, ^51^, ^52^, ^53^, ^54^, ^55^ There have been indications of a worse prognosis due to the occurrence of the latter,^37^, ^38^, ^42^, ^43^, ^54^, ^56^, ^57^ but their clinical significance remains controversial.^32^, ^38^, ^39^, ^44^, ^45^, ^49^, ^50^, ^51^, ^55^, ^58^ In addition, the methodology of detection varies considerably between these studies. It seems problematic to deduce a pragmatic guide to processing LNs by this. Why should we use more and more resources for a result of uncertain value?

It may be argued that the UICC classification does not suggest an extensive analysis and thus conclude that a single H&E‐stained section of an LN is enough. This also seems problematic, considering the possibility and a certain probability of missing metastasis.^48^ What physicians want to consciously blind themselves when it comes to diagnostics? Taking this into account, the results of our study reveal a number of interesting facts and will be useful in determining an appropriate approach to this issue.

Complete stepwise sectioning of the LNs detected by the SP revealed more metastases (69 vs. 77). This difference was not significant. When taking into account the additional and not grossly visible LNs obtained by stepwise sectioning of the blocks, the number of detected metastases increased to 80, which differed significantly from the SP number. This means that the increase in metastases was proportional to the number of additional LNs and not due only to finding new metastases in the LNs already seen in the SP. Nevertheless, these additional LNs were found by sectioning paraffin blocks. Therefore, the method of stepwise sectioning has an effect on statistics. However, this significant increase in metastases did not influence staging or treatment. Only in cases where there were already metastases detected by the SP were new metastases found by use of the EP.

This statement was confirmed by the use of immunohistochemistry in the second study group. Staining for CK (AE1/AE3) revealed more metastases (82 vs. 89), but only in patients in whom metastases had already been seen in the conventional H&E section. Here too, there was an effect on statistics but not on staging.

Furthermore, when there was a metastasis in a distant site, there was also at least one metastasis in LNs near the tumour. This is not surprising, but it is important for sufficient workup of the surgical specimens, as 54% of LNs and 93% of metastases are seen in a 10‐cm colon segment around the tumour. Therefore, when it is necessary to look for additional LNs and metastases, it is more efficient to work up paraffin blocks with the fat taken from near the tumour. A very similar result was already shown by Mescoli *et al*.^53^, ^54^

Moreover, it is not helpful to embed LNs completely if the diameter is >5 mm. In 3/80 LNs, the metastasis was found in only one half of the node. In all these three particular cases, metastases were also detected in other LNs. Thus, embedding both LN halves did not provide more relevant information.

Isolated tumour cells were explicitly excluded from the count of metastases in our investigation, but their appearance should be briefly mentioned. In two cases in Group 1 and one case in Group 2, isolated tumour cells not previously detected in the SP were detected in the EP. In all three of these cases, valid metastases according to the TNM definition of >0.2‐mm extent had already been found by the SP.

Concerning tumour deposits as another special situation of classification, we recorded a single one in a case in Group 1. The pN1c category assigned in the SP did not change after using the EP.

Incidentally, no clear correlation could be seen between the tumour entity or tumour grading and metastases that were not detected by the SP. In total, from the two study groups there were 14 cases with additional metastases detected by the EP. In 12 of these 14 cases (86%) the tumour was classified as ‘adenocarcinoma NOS, G2’, according to the WHO Classification of Tumours: Digestive System Tumours, 5th Edition. The two remaining cases fall into the categories ‘undifferentiated carcinoma, G4’ and ‘mucinous adenocarcinoma, G2’.

It has also to be mentioned, that the overall number of LNs found was not important in regard to these findings. When parting Group 1 in two subgroups with <20/>19 LNs, this did not change findings. In this context it has to be said, that the number of 12 LNs needed for correct UICC classification is a minimum, not a mean. Reaching 12 LNs does not mean to stop careful examination of pericolic fat. This is a basic part of the work and pathologist are responsible for correct evaluation.^59^, ^60^, ^61^

We were also unable to identify any significant difference between right‐ and left‐sided cases. It should be noted, however, that a considerably higher number of right‐sided cases were acquired. In this context, it can be assumed that the results reported so far are valid regardless of the tumour location.

Whether these findings are applicable to neoadjuvantly treated rectal carcinomas remains to be seen in future studies, but is likely. Given the requirement for thorough histological examination even in such cases, any potential savings in processing time would be important^62^.

Overall, the EP was not superior to the SP for defining the appropriate UICC staging. In none of the 50 cases analysed in Group 1 and in none of the 55 cases analysed in Group 2 did the EP lead to a change in staging or treatment, and only minor changes in the pTNM classification were detected. Also, the EP increased the time needed to examine additional tissue sections many times over without any patient benefit and with increased costs.

In conclusion, the SP described above is in concordance with the UICC classification without loss of clinically relevant information. Additional metastases are found only if metastases were already detected. The sample size of our study allowed us to come to this conclusion, which has to prove itself in future investigations.

## Author contributions

Conception: Ludwig Wilkens, Moritz Rust, Nabih Farkouh, Ali Al‐Mukahal. Administrative support: Clara Böker, Julian Mall, Ludwig Wilkens. Collection of data: Moritz Rust, Nabih Farkouh, Ali Al‐Mukahal, Clara Böker, Julian Mall. Data analysis and interpretation: Piet Beusker, Moritz Rust, Nabih Farkouh, Ludwig Wilkens. Manuscript writing: Moritz Rust, Nabih Farkouh, Ludwig Wilkens. Final approval of manuscript: All authors.

## Conflict of interest

The authors declare that they have no conflict of interest in the study conducted.

## Funding information

The authors declare that they did not receive any third‐party funding for the study.

## Data Availability

The authors declare that the data collected in the study may only be used after direct consultation with them.
